# Navigating Innovation Research: Practical Strategies for Early-Career Scholars

**DOI:** 10.1093/geroni/igaf122.1379

**Published:** 2025-12-31

**Authors:** Lillian Hung

**Affiliations:** University of British Columbia, Vancouver, British Columbia, Canada

## Abstract

Integrating new technologies into long-term care (LTC) presents exciting opportunities and significant challenges. As the aging population grows, there is increasing interest in how artificial intelligence (AI)-enabled robots can support residents’ well-being and improve care delivery. Drawing on my research with AI-powered social robots such as Aether, Paro, and Lovot, this presentation examines the practical considerations of adopting these innovations in LTC. Robotic technologies offer many benefits, including enhanced engagement, cognitive stimulation, companionship, and reduced loneliness. These tools may also help alleviate caregiver burden by assisting with repetitive tasks or providing entertainment and therapeutic interactions. However, implementation presents challenges. Ethical concerns—such as ensuring equitable access, avoiding substitution of essential human care, and preserving dignity and autonomy—must be carefully navigated. Practical barriers, including staff training, infrastructure adjustments, cost, and acceptance by residents and care providers, also affect successful adoption. This presentation highlights key lessons learned and offers strategies for conducting successful innovation research. Early-career scholars should seek diverse funding sources, secure pilot study grants, and align projects with practice needs. Networking within aging, technology, and healthcare communities fosters mentorship, partnerships, and knowledge exchange. Publishing in both academic and practitioner-oriented outlets bridges research and real-world application. By ensuring responsible implementation, AI-enabled robots can be used ethically and effectively to support resident well-being, promote person-centered care, and complement rather than replace human interactions in LTC settings.

